# Type I insulin-like growth factor receptor signaling in hematological malignancies

**DOI:** 10.18632/oncotarget.12123

**Published:** 2016-09-19

**Authors:** Deeksha Vishwamitra, Suraj Konnath George, Ping Shi, Ahmed O. Kaseb, Hesham M. Amin

**Affiliations:** ^1^ Department of Hematopathology, The University of Texas MD Anderson Cancer Center, Houston, TX, USA; ^2^ State Key Laboratory of Bioreactor Engineering, East China University of Science and Technology, Shanghai, China; ^3^ Department of Gastrointestinal Medical Oncology, The University of Texas MD Anderson Cancer Center, Houston, TX, USA; ^4^ The University of Texas Graduate School of Biomedical Sciences, Houston, TX, USA

**Keywords:** IGF-IR, plasma cell myeloma, leukemia, lymphoma

## Abstract

The insulin-like growth factor (IGF) signaling system plays key roles in the establishment and progression of different types of cancer. In agreement with this idea, substantial evidence has shown that the type I IGF receptor (IGF-IR) and its primary ligand IGF-I are important for maintaining the survival of malignant cells of hematopoietic origin. In this review, we discuss current understanding of the role of IGF-IR signaling in cancer with a focus on the hematological neoplasms. We also address the emergence of IGF-IR as a potential therapeutic target for the treatment of different types of cancer including plasma cell myeloma, leukemia, and lymphoma.

## INTRODUCTION

Several major advances have improved our understanding of the molecular characteristics of hematological malignancies and led to the development of new therapeutic agents to eliminate these aggressive neoplasms. The discovery of the *BCR-ABL1* fusion oncogene and defining the pathogenetic molecular mechanisms in chronic myelogenous leukemia (CML) have led to the development of BCR-ABL tyrosine kinase inhibitors such as imatinib, nilotinib, and dasatinib, which have improved the 10-year survival rate drastically in CML patients, from 20% to 85% [1–5]. In patients diagnosed with indolent or aggressive B-cell non-Hodgkin's lymphoma, the use of the anti-CD20 antibody rituximab has resulted in improved survival [6]. These are only some of the most recognized examples of the breakthroughs that have occurred in the field of developing new therapies to treat hematological neoplasms. In spite of these discoveries, patients diagnosed with hematological malignancies continue to experience disease relapse and resistance to available treatment options, which suggests that the need to develop novel approaches that can be used alone or in combination with current therapeutic modalities to eradicate hematological neoplasms remains critical.

Numerous studies have concluded that the type I insulin-like growth factor receptor (IGF-IR) and its primary ligand IGF-I play significant roles in the establishment and progression of tumors, primarily by inhibiting apoptosis and inducing cellular transformation [7–10]. IGF-IR is also thought to aid malignant cells in acquiring anchorage-independent growth, giving the cells the ability to survive detachment and facilitate migratory processes for metastatic dissemination [11].

To date, there are several potentially effective IGF-IR inhibitors that have been tested in preclinical studies as well as in clinical trials enrolling patients harboring aggressive forms of solid cancers and hematological malignancies. Importantly, these IGF-IR inhibitors are well tolerated with minimal toxic effects *in vivo* [12]. The effects of IGF-IR have been studied to a great extent in solid tumors, including those of the breast, prostate, lung, ovary, skin, and soft tissues [13–17]. In contrast, less studies have been performed to thoroughly examine the function of IGF-IR in hematological neoplasms [18–24]. In this review, we discuss the current understanding of the role of IGF-IR signaling in cancer including hematological neoplasms. We also address the emergence of IGF-IR as a potential therapeutic target in the treatment of these aggressive diseases.

## THE IGF SIGNALING SYSTEM

### Overview

**Figure 1:**
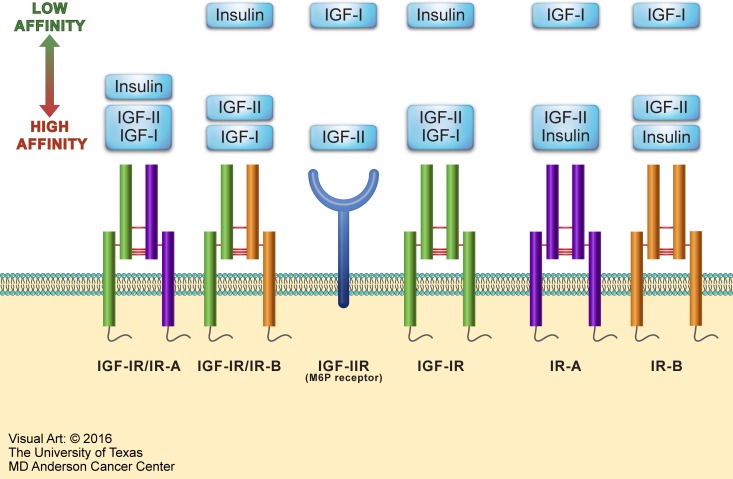
Overview of the IGF system The IGF system consists of four receptors: IR, IGF-IR, IGF-IIR, and hybrid receptors. IR is expressed as two isoforms - IR-A and IR-B. IR-A has oncogenic potential, expressed predominantly in fetal tissues, and its expression declines during adulthood. IR-B is the physiologically expressed isoform in adult tissues. The IR-A or IR-B receptor makes one half of the hybrid receptors along with one half of the IGF-IR. The IGF system receptors interact mainly with three ligands: insulin, IGF-I, and IGF-II. Excluding IGF-IIR, these receptors possess tyrosine kinase activity. At the other hand, IGF-IIR (also known as mannose-6-phosphate [M6P] receptor) binds and removes circulating IGF-II to keep its free form at very low levels. The figure depicts IGF system ligands in order of their binding affinities to the different receptors. Ligands shown within the same rectangle have almost similar affinities to bind with a specific receptor. Ligands shown in separate yet close rectangles have slightly different receptor binding affinities. When the rectangles are widely separated, the ligands binding affinities are remarkably different.

The IGF signaling system plays significant roles in both embryonic and postnatal development as well as having important functions in normal adult physiology. The IGF system includes four receptors: insulin receptor (IR), IGF-IR, IGF-IIR, and the hybrid receptors consisting of one-half IR and one-half IGF-IR (Figure 1). These receptors interact with three main ligands: insulin, IGF-I, and IGF-II. IR, IGF-IR, and IGF-IIR have the strongest binding affinity for their respective ligands, whereas the binding of insulin to IGF-IR and IGF-I to IR is at least 100-fold less [25]. IGF-I and IGF-II signaling is mediated through IGF-IR; but IGF-I has at least 3-fold higher binding affinity than does IGF-II [25]. The IGF system also includes regulatory proteins, known as IGF binding proteins (IGFBPs) that regulate IGF signaling. Although up to 10 proteins have been described in the literature as IGFBPs, only IGFBP-1 thorough IGFBP-6 are considered true IGFBPs based on their conserved protein structure and high binding affinity for IGF-I and IGF-II [26].

### IGF-IR

**Figure 2:**
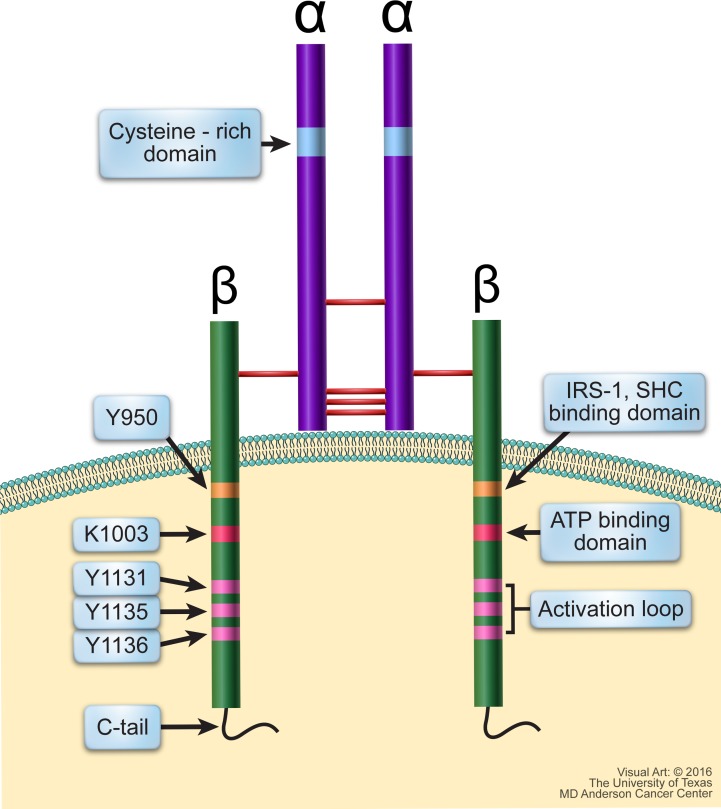
Schematic structure of IGF-IR IGF-IR is a transmembranous homodimeric receptor tyrosine kinase that is composed of two identical extracellular α subunits and two identical transmembranous β subunits connected by disulfide bonds. The α subunit contains a cysteine-rich domain where the ligand binds. The cytoplasmic regions of the β subunits contain residues directly involved in IGF-IR signaling. Y950 is the binding site for its primary substrates including IRS-1 and SHC; Lys1003 is the ATP binding domain; and Tyr1131, Tyr1135, and Tyr1136 make up the activation loop of the kinase domain, which is followed by the C-terminus domain.

IGF-IR is a receptor tyrosine kinase that is structurally composed of two identical α subunits and two identical β subunits that are connected by disulfide bonds to form the functional homodimeric receptor complex (Figure 2). Each α subunit is entirely extracellular and contains a cysteine rich domain that forms the primary binding site for its ligands IGF-I, IGF-II, and, to a much lesser affinity, insulin. Each β subunit includes an extracellular domain, a 24-residue hydrophobic transmembrane domain, and a larger cytoplasmic region that shares 84% amino acid sequence identity with the IR [27]. Tyrosine 950 is the binding site for its substrates IRS-1 and SHC, among others. The intracellular region of the β subunit contains an ATP binding site at lysine 1003. It also contains a kinase domain encompassing the activation loop made up of three critical tyrosine residues at positions 1131, 1135, and 1136, which form part of the Y*XXX*YY moiety (Y is a tyrosine and X is a non-tyrosine amino acid residues). It is important to note that the Y*XXX*YY moiety is also present in other members of the IR family such as the anaplastic lymphoma kinase (ALK) and leukocyte tyrosine kinase receptors [28–30]. The tyrosine residues within the Y*XXX*YY moiety become phosphorylated upon ligand binding-induced receptor dimerization. Residue 1136 in IGF-IR is particularly important in that it maintains the conformational stability of the β subunit.

**Figure 3:**
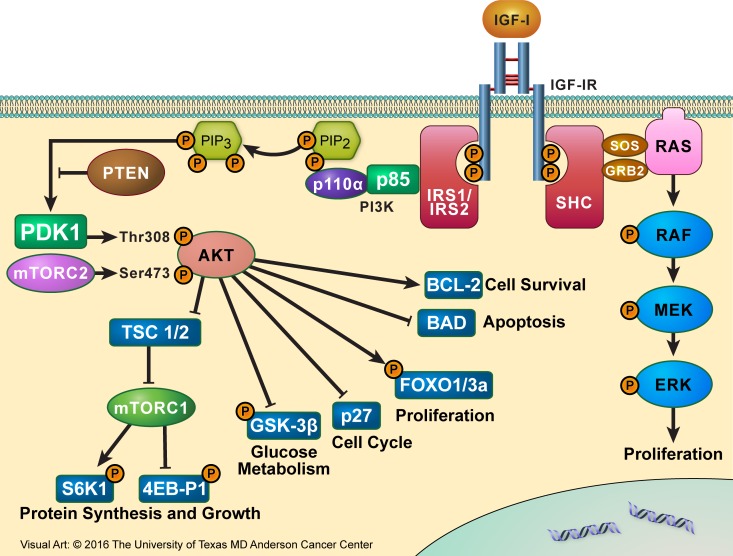
IGF-IR signaling partners Binding of IGF-IR to its primary ligand, IGF-I, causes the phosphorylation of tyrosine residues present in the intracellular portion of its β subunit. Once phosphorylated, Y950 provides a docking site for its substrates IRS-1-4 and SHC proteins. Upon substrate binding, downstream signaling is activated through the IRS-1/PI3K/AKT/mTOR or SHC/RAS/MAPK pathway, leading to proliferative and anti-apoptotic effects, which culminate to oncogenesis and tumor cell survival.

The C-terminus domain of IGF-IR contains several tyrosine and serine residues. These residues are phosphorylated, probably to induce mitogenic effects, but how their phosphorylation actually contributes to normal and malignant IGF-IR signaling is still not fully understood. The binding of IGF-IR to its ligands causes the phosphorylation of tyrosine residues located in the intracellular portion of its β subunit. Once phosphorylated, tyrosine 950 provides a docking site for IGF-IR substrates including IRS-1-4 and SHC proteins, which, in turn, act as docking sites. Upon substrate binding and phosphorylation of docking sites, downstream signaling is activated through the PI3K/AKT, MAPK, and STAT pathways and may stimulate cancer cell growth in an autocrine/paracrine manner [31–33] (Figure 3).

Mouse models have revealed the importance of IGF-IR in prenatal and postnatal growth, especially in regards to genetic imprinting. The activation of IGF-IR during these stages improves the survival and increases the proliferation of mitosis-competent cells, resulting in the growth of tissues such as skin, bone, adipose tissue, and skeletal and cardiac muscles [34–38]. IGF-IR also plays roles related to the development of the mammary gland during the embryonic stage. In this regard, IGF-IR regulates the formation and proliferation of terminal end buds and contributes to ductal outgrowth and branching during puberty. In addition, it has been demonstrated that IGF-IR is necessary for normal epithelial proliferation and alveolar formation during pregnancy and lactation [39]. During embryonic development, the IGF-IR pathway is also involved in the development of limb buds. It has been previously shown that the abrogation of IGF-IR expression such as in *Igf1r*-null mice or the marked decrease in IGF-I levels leads to generalized organ hypoplasia, including developmental delays in bone ossification and abnormalities in the central nervous system [35, 38, 40–43]. Mice lacking the entire *Igf1r* gene typically exhibit a dramatic reduction in body mass, and they die at birth due to severe respiratory failure [40]. Mice carrying only one functional copy of *Igf1r* are born alive, but their body mass is ~45% lower than normal [40].

The aberrant activation of the IGF-IR pathway is also strongly associated with initiating cancer growth [12]. Within the past 20-30 years, evidence has emerged to support that IGF-IR overexpression plays a significant role in the development and progression of tumors, metastatic potential, and resistance to therapies. Studies have shown that IGF-IR induces its oncogenic effects through the inhibition of apoptosis and the induction of transformation and angiogenesis. Furthermore, IGF-IR regulates properties that cause malignant cells to acquire anchorage-independent survival, allowing them to endure detachment and migration, which is essential for metastatic dissemination [44].

Moreover, it has been documented that IGF-IR expression above a certain threshold can induce tumor cell progression that is independent of exogenous ligand stimulation. For example, when RM11A murine breast cancer cells that express high levels of exogenous IGF-IR were treated with IGF-I or IGF-II, the proliferative/survival potential of these cells was not enhanced, suggesting a ligand-independent mechanism [45]. At the other hand, wild-type RM11A cells expressing low levels of IGF-IR had a robust response to IGF stimulation, most likely because the activation of endogenously expressed low levels of IGF-IR is still dependent on IGF-I stimulation. Therefore, it was proposed that high expression of IGF-IR results in its constitutive activation independent of the presence of ligands [45].

It has also been shown that the level of IGF-IR expression, specifically a minimum number of its molecule, plays a key role in its transforming and oncogenic properties [46, 47]. For instance, in 3T3-like fibroblasts, the IGF-IR number needs to be greater than 20,000 receptors per cell to enable mitogenesis and transformation after stimulation with IGF-I [46]. Similarly, in a pancreatic cell line, PANC-1, the observed increase in the receptor number per cell from 40,000 to 100,000 is in the range required to enable IGF-I-stimulated growth and therefore may be of central importance for pancreatic tumor growth [47]. Based on these findings, it was concluded that a relatively small increase in the number of receptors per cell above a certain threshold could induce the transformation effects of IGF-IR.

Studies on IGF system in normal epithelial and cancerous cells such as those of the breast have found that IGF-IR is activated in one of two ways through IGF-I: (1) hepatic production of IGF-I can increase serum IGF-I, which in turn may act in an autocrine manner as a stimulatory molecule to induce the proliferation of normal as well as cancer cells; and/or (2) local production of IGF-I by stromal cells that surround the tumorigenic cells serves as a paracrine stimulator of the IGF-IR signaling [48, 49]. It has also been proposed that the interactions between IGF-I and IGF-IR enhance tumor cell survival through stimulation of other oncogenic signaling mechanisms. For instance, in prostate cancer cells, IGF-I is thought to indirectly stimulate the androgen receptor via crosstalk with IGF-IR to induce the transformation of prostatic epithelial cells [50, 51]. The oncogenic effects of IGF-I/IGF-IR interactions have also been suggested to exist in colon cancer. IGF-IR is highly expressed in colon cancer, compared to normal colonic mucosa, and IGFs in colonic malignant mucosa could exert their effects via IGF-IR in a paracrine manner [51–53].

In contrast to the widely studied solid tumors, not many studies have examined the role of IGF-IR in hematological neoplasms, and most of these studies focused on plasma cell myeloma [54, 55]. Other studies also addressed the role of IGF-IR signaling in acute myelogenous leukemia (AML) and acute lymphoblastic leukemia (ALL) [56–64]. In addition, studies from our lab and others' have demonstrated the contributions of IGF-IR signaling to the pathogenesis of other hematological neoplasms, including T-cell lymphoma, mantle cell lymphoma, chronic lymphocytic leukemia (CLL), and CML [20–24, 65].

### IGF-IIR

Also, there are studies showing the involvement of IGF-IIR in cancer development and progression. IGF-IIR, known as the cation-independent mannose-6-phosphate receptor, acts as a reservoir to clear IGF-II from the extracellular environment and cell surface through endocytosis and lysosomal degradation, thereby inhibiting IGF-II-induced tumor growth signaling [66–68]. Therefore, IGF-IIR is considered a tumor suppressor protein. It has been demonstrated that specific alterations in *IGF-IIR* lead to the progression of a transformed phenotype. For example, microsatellite instability of *IGF-IIR* has been documented in cancers of the prostate, breast, endometrium, and gastrointestinal tract [69–71]. In addition, loss of heterozygosity of *IGF-IIR* has been described in liver, lung, ovarian, prostate, head and neck, and breast cancers [72–76]. While loss of heterozygosity in tumor samples suggests the loss of *IGF-IIR* function and the enhancement of the progression toward a transformed phenotype, the detection of mutations in *IGF-IIR* that disrupt ligand binding at the cell surface is another mechanism by which *IGF-IIR* loses its function [77, 78].

### IGF-I and IGF-II

IGF-I is a single-chain peptide that shares 62% and 40% amino acid sequence identities with IGF-II and proinsulin, respectively [79]. IGF-I is synthesized in a variety of tissues and cultured cell types, suggesting that this protein may have autocrine and paracrine effector functions, unlike insulin, which is stored within cells of a particular tissue (pancreas) and has mainly autocrine effects [38, 80]. Liver secretion of IGF-I is regulated by the growth hormone (GH), which signals peripheral tissues to grow, whereas insulin is tightly regulated by glucose uptake [80, 81]. Therefore, insulin is primarily associated with metabolic effects, whereas IGF-I is essentially a growth factor and an anabolic agent.

IGF-I is a ~7.6 kDa 70 amino acid single chain polypeptide that functions as the primary ligand for IGF-IR. There is increasing evidence that IGF-I might provide a major link between IGF-IR and the development of cancer through its regulatory effects on cell proliferation, differentiation, and apoptosis. Although cancer cells do not necessarily secrete IGF-I, high concentrations of circulating IGF-I, which is secreted by the liver, have been found to associate increased cancer risk in children and adults as well as to contribute to the growth, maintenance, and progression of cancer [82–86]. Mouse models have shown reduced proliferation of androgen-dependent prostate cancer cells in IGF-I-deficient hosts relative to control hosts [87]. In contrast, spontaneous neoplasia occurs in prostate epithelium from *Igf1* transgenic mice [88]. In breast cancer, it has been shown that elevated IGF-I levels can reduce the synthesis of sex hormone-binding globulin, which can lead to an increase in the bioavailability of estrogen. Estrogen has been demonstrated to induce the expression of IGF-IR as well as IRS-1 and IRS-2, which synergistically potentiate the activation of MAPK [89].

IGF-II is a ~7.5 kDa 67 amino acid single chain mitogenic polypeptide that is produced mainly by the liver [68, 90–93]. It is also synthesized by other tissues, such as muscle and placenta, in which it elicits its effects through an autocrine or paracrine manner, similar to IGF-I [94, 95]. Physiologically, IGF-II is involved in the regulation of fetal development (it is highly expressed during embryonic stages), cell growth, differentiation, and metabolism [90, 96–104]. Unlike IGF-I that is tightly regulated by GH, genetic factors, such as loss of imprinting, play a significant role in the regulation of *IGF-II,* which causes an overabundance of IGF-II and subsequent augmentation of its signaling [105, 106]. Loss of imprinting has been demonstrated in a variety of cancers including breast, ovarian, and esophageal, and kidney cancers. Furthermore, *IGF-II* can also be regulated at the transcriptional level either through direct binding of transcription factors on consensus motifs or through hormone-induced transcription [107–112]. IGF-II is believed to induce its effects mainly through IGF-IR, IR isoform A (IR-A), and IGF-IR/IR-A hybrid receptors. Similar to IGF-I, the binding of IGF-II to IGF-IR induces downstream signaling via the PI3K/AKT and MAPK pathways [113]. IGF-II is cleared from the extracellular environment through binding with IGF-IIR. The overexpression of IGF-II in cancer cells has been demonstrated using *in vitro* and *in vivo* experimental approaches as well as in cancer patients' specimens [114–120]. Moreover, *in vivo* studies showed that IGF-II overexpression results in more aggressive tumors, while animals with low expression of IGF-II or treated with IGF-II-neutralizing antibodies had lower tumor burdens and prolonged survival [114, 117, 118, 121].

## ROLE OF IGF-IR SIGNALING IN HEMATOLOGICAL MALIGNANCIES

### Plasma cell myeloma

Plasma cell myeloma (PCM) accounts for approximately 10% of hematological malignancies with an incidence of four cases per 100,000 people a year [122]. The average age at diagnosis is 68 years, and the incidence increases with age advancement. The development of PCM occurs in steps. Normal plasma cells are usually arrested in the G_1_ phase of the cell cycle; however, malignant PCM cells exhibit uncontrolled cell cycle progression. PCM cells initially reside in the bone marrow, where they interact with stromal cells that express integrins and secrete cytokines to potentiate migration, adhesion, and proliferation of the PCM cells, and enhance surrounding angiogenesis [123–125]. The plasma cells then ultimately invade the basement membrane of the bone marrow and may undergo widespread dissemination. These processes are thought to be highly dependent on interleukin-6 (IL-6), as targeting IL-6/IL-6 receptor (IL-6R) signaling decreases PCM tumor growth *in vitro* and *in vivo* [126–129].

In addition to IL-6, it has also been shown that IGF-I induces the growth and development of PCM through activation of IGF-IR. For instance, some earlier studies demonstrated that IGF-I is capable of increasing DNA synthesis as well as enhancing the viability and proliferation of several PCM cell lines. The effects of IGF-I were reversed when an anti-IGF-IR blocking antibody was additionally used [130, 131]. In further support of an important role of IGF-I/IGF-IR signaling in PCM, studies from different labs showed that IGF-IR and IGF-I are highly expressed in PCM, and that the IGF-I/IGF-IR signaling axis, at least partially through an autocrine cascade, contributes significantly to the survival and progression of PCM [54, 132]. Different models have been proposed to explain the functional relationship between IGF-I/IGF-IR and IL-6 in PCM. In one model, the effects of IGF-I and IL-6 on PCM cells appeared to be executed independently. Indeed, anti-IGF-IR/IGF-I antibodies failed to block the stimulatory effects of IL-6. In a similar fashion, anti-IL-6/gp130 antibodies were not capable of reversing the effects of IGF-I [133]. Importantly, IGF-I and IL-6 were found to induce “additive” effects on PCM cell survival and proliferation. In support of this idea, IGF-I/IGF-IR axis induced its effects predominantly through the phosphorylation of IRS-1 protein, which is considered a major substrate of IGF-I/IGF-IR signaling [31, 134, 135]. Moreover, IL-6/gp130 promoted the survival of PCM cells through the phosphorylation of STAT3. Both signaling pathways, however, efficiently activated the RAS/ERK/MAPK survival-promoting system [133]. In another model, IL-6 was found to phosphorylate/activate IGF-IR receptor in PCM cell lines [136]. Interestingly, IL-6 stimulation of PCM cells was associated with co-localization of the IL-6Rα with IGF-IRβ at lipid rafts.

IGF-I/IGF-IR axis supports the survival of PCM cells not only via signaling through IRS-1 but also by singling through IRS-2. The IRS-2/PI3K/p70S6K pathway was found to be either constitutively activated or instead activated via upstream interactions with IGF-I/IGF-IR [19]. When IRS-2/PI3K/p70S6K pathway is constitutively activated, the direct binding of IRS-2 with IGF-IR, regardless of IGF-I stimulation, induces downstream effects that lead to tumor cell proliferation and inhibition of apoptosis [19].

### IGF-IR signaling inhibits apoptosis and induces proliferation of PCM cells

In PCM, IGF-I/IGF-IR signaling inhibits apoptosis and induces tumor cell proliferation through the activation of two distinct pathways - PI3K/AKT and MAPK. Which of the two processes - apoptosis or cell proliferation - is promoted appears to depend on which pathway is activated. For instance, IGF-IR suppresses apoptosis through inhibition of the release of cytochrome C from the mitochondria after the activation of the PI3K/AKT pathway. Activated AKT phosphorylates the pro-apoptotic protein BAD. Upon phosphorylation, BAD becomes dissociated from anti-apoptotic BCL-2 and sequestered by the adaptor protein 14-3-3. These processes prevent apoptosis from occurring in PCM cells [137, 138].

One possible mechanism for the activation of the PI3K/AKT pathway in PCM is the lack of the PTEN tumor suppressor protein, a negative regulator of PI3K/AKT activity [139, 140]. The anti-apoptotic effect of PI3K/AKT signaling after IGF-I stimulation was found to be reversed upon overexpression of PTEN in PCM cell lines lacking PTEN, suggesting that the loss of PTEN is also responsible for uncontrolled IGF-IR-induced AKT activity in these cells [139, 140].

Whereas the activation of the PI3K/AKT pathway by IGF-IR appears to be involved primarily with inhibition of apoptosis in PCM, activation of the MAPK pathway is believed to be principally involved with inducing cell proliferation [138]. Ligand binding of IGF-I to IGF-IR allows the phosphorylation of the SHC substrate, which in turn leads to activation of RAS that subsequently activates the RAF kinase on its serine/threonine residues. As a result, activated RAF phosphorylates and activates MEK, which then phosphorylates and activates MAPK, leading to the proliferation of PCM cells [141].

In addition to the idea that distinct outcomes result from IGF-IR-induced activation of PI3K/AKT versus IGF-IR-induced activation of MAPK, more recent studies have revealed that “crosstalk” exists between PI3K/AKT and MAPK and that this crosstalk is critical for PCM tumor progression [141, 142]. In this model, binding of IGF-I to IGF-IR induces phosphorylation of RAF, MEK, and MAPK. However, inhibition of PI3K using the LY294002 inhibitor resulted in the inhibition of MEK and MAPK without interfering with the activation of upstream RAF [141]. This suggests that the PI3K pathway is capable of regulating the activation of MAPK, bypassing upstream activators of this pathway. Alternatively, using the MAPK inhibitor PD98059 did not have significant effects on the PI3K pathway, alluding to the idea that this crosstalk is mediated by PI3K, and not vice versa.

In a similar fashion, it was shown that rapamycin-induced mTOR inhibition decreased the serine phosphorylation of IRS-1, which was associated with a compensatory IGF-I downstream signaling via the PI3K/AKT pathway [142]. It was noted that this activation is highly dependent on IGF-IR signaling, because treating the PCM cells with an anti-IGF-IR antibody hindered the ability of rapamycin to activate AKT. Related observations were also shown in a different study utilizing the IGF-IR tyrosine kinase inhibitor picropodophyllin (PPP), where it was found that PPP significantly sensitized RPMI 8226 PCM cells to rapamycin and SB203580, a MAPK inhibitor [143]. These observations suggest a relationship and possible crosstalk between the mTOR and IGF pathways in PCM [142, 144].

In addition to the effects mediated by IGF-I, *in vitro* experiments have shown that IGF-II increases cell proliferation and protects dexamethasone-treated PCM cells from apoptosis [145]. Although this study did not examine in detail the interactions between IGF-II and IGF-IR, it was implied that at least the IGF-II-mediated proliferation of PCM cells was executed through IGF-IR. Whether the anti-apoptotic effects of IGF-II were also mediated through IGF-IR remains to be elucidated. Notably, IGF-II was almost as effective as IGF-I in protecting the PCM cells from dexamethasone-induced apoptosis [145].

### IGF-IR induces adhesion, invasion, and migration of PCM cells

IGF-I/IGF-IR signaling is also involved in critical aspects of PCM dissemination, including adhesion, invasion, and migration. One of the principal features of malignant PCM cells during homing is their ability to transmigrate and adhere to the bone marrow microenvironment. In this regard, IGF-I acts as a chemo-attractant for PCM cells that increases their adhesion to the extracellular matrix glycoprotein fibronectin [146]. This effect is also dependent on the activation of IGF-IR downstream targets including PI3K/AKT [146]. In addition, IGF-I can rapidly and transiently induce the association between IGF-IR and β_1_ integrin, a heterodimeric membrane protein that mediates cell adhesion to the extracellular matrix, which also leads to further adhesion of PCM cells to fibronectin [146]. The phosphorylation of IGF-IR, IRS-1, and PI3K leads to the co-localization of IGF-IR and β_1_ integrin on lipid rafts in the cell membrane [146, 147].

It has also been shown that IGF-I triggers the polymerization of F-actin, induces phosphorylation of FAK and paxillin, and enhances β_1_ integrin's association with these focal adhesion proteins, which enhances PCM homing to the bone marrow microenvironment [146, 148]. IGF-I induces the migration of PCM cells in an AKT-dependent or -independent manner, such as through the PKD or RhoA/PKC pathway [146, 149, 150]. This was further demonstrated when an anti-IGF-I antibody, anti-β_1_ integrin antibody or PI3K inhibitor abrogated IGF-I-induced transmigration [146].

### Acute and chronic leukemia and myelodysplastic syndromes

Upregulation of IGF-I/IGF-IR signaling is prevalent in several types of leukemia, including AML, B-ALL, T-ALL, CML, and CLL.

Overexpression of IGF-IR has been reported in human AML cells and autocrine IGF-I production has been suggested to play a role in drug resistance in AML [18, 56, 57]. Furthermore, constitutive activation of the PI3K/AKT signaling pathway is believed to play crucial roles in the survival of AML cells [151, 152]. Constitutive activation of PI3K/AKT, at least in part, results from autocrine IGF-I release and activation of IGF-IR, which was shown in 70% of AML samples [18, 57]Several *in vitro* studies using different IGF-IR inhibitors illustrated the therapeutic potential of targeting IGF-IR in AML. For example, NVP-ADW742, a small molecule inhibitor of IGF-IR, induces AKT dephosphorylation, which subsequently decreases p38 phosphorylation and downregulates antiapoptotic proteins such as BCL-2 in AML cells [60]. Inhibition of constitutive IGF-IR phosphorylation as well as downstream signaling through MEK and AKT was also demonstrated after treatment with BMS-536924 or BMS-554417, dual IGF-IR/IR inhibitors [59, 153]. In another study, the IGF-IR small molecule inhibitor NVP-AEW541 sensitized primary AML blasts and cell lines to etoposide-induced apoptosis through caspase-3 cleavage, in addition to inducing antiproliferative effects [56].

In addition, in AML, it has also been shown that mTOR inhibition using rapamycin overactivates PI3K/AKT by upregulating IGF-I/IGF-IR signaling, in a fashion similar to that seen in PCM. These effects appear to occur through enhancing not only IRS-1 expression but also IRS-2 expression. Using 19 AML samples with constitutive PI3K/AKT activation, the rapamycin-derivative inhibitor everolimus increased AKT phosphorylation by upregulating the expression of IRS-2 via an IGF-I/IGF-IR autocrine loop [58]. These findings suggest crosstalk between IGF-IR, PI3K/AKT, and mTOR in AML.

The myelodysplastic syndromes (MDS) arise from clonal hematopoietic stem cells that are characterized by atypical morphology, aberrant maturation, and specific molecular events. Approximately, one third of MDS patients experience progression to AML [154]. Decreased apoptosis of the hematopoietic cells plays an important role in the pathogenesis of MDS and probably in its transformation to AML [155–157]. Recently, IGF-IR has been shown to be involved in MDS pathogenesis and its transformation to AML. Compared with normal myeloid cells, IGF-IR protein and mRNA expression is enhanced in MDS cells, and this enhancement increases with progression to AML. In addition, in both MDS and AML, apoptotic signals are present only in IGF-IR-negative cells, suggesting that IGF-IR protects these cells from apoptosis [158].

Our group has discovered that IGF-IR also plays a role in CML [21]. In our study, IGF-IR was upregulated in CML cell lines, and the expression of IGF-IR appeared to be directly correlated with the progression of CML. For instance, IGF-IR was expressed in 30% and 25% of patients with chronic phase and accelerated phase CML, respectively, but the expression rate increased to 73% in patients with blast phase CML. Treatment of CML cells with PPP, an IGF-IR small molecule inhibitor, resulted in decreased cell viability and proliferation. In addition, inhibition of IGF-IR decreased activated/phosphorylated AKT and STAT5 as well as downregulated BCL-2, BCL-X_L_, and basal caspase-3 and was associated with apoptotic cell death. Inhibition of IGF-IR also resulted in cell cycle arrest at the G_2_/M-phase, which was mediated through upregulation of cyclin B1 and downregulation of cyclin E and pCdc2 [21].

In another recent study, the involvement of c-myb with IGF system in inducing the proliferation of CML cells was demonstrated [159]. C-myb plays an important role in the regulation of cell growth and differentiation and is highly expressed in malignant hematopoietic cells. Induced overexpression of c-myb causes a significant increase in IGF-I, IGF-II, and IGF-IR expression, and a decrease in IGFBP-3 expression, which promotes CML cell proliferation [159]. Of note is that anti-IGF-IR antibodies inhibited the c-myb-induced cell growth. The growth-promoting effects of c-myb were mediated through two major intracellular signaling pathways, AKT and ERK. The activation of AKT and ERK by c-myb was abrogated by IGF-IR and IGFBP-3 antibodies. These findings suggest that c-myb stimulates cell growth, in part, by regulating the expression of the components of the IGF system in CML.

The contribution of IGF-I/IGF-IR signaling to CLL has also been investigated. It was shown that IGF-I and IGF-IR participate in autocrine/paracrine loops to promote the survival of CLL cells [160]. High levels of IGF-IR protein and mRNA expression were detected in CLL cells and positively correlated with the expression of the antiapoptotic protein BCL-2. It was also shown that serum IGF-I was elevated in CLL patients, but the level of GH remained unchanged, suggesting that autocrine production of IGF-I by CLL cells results in increased levels of serum IGF-I, independently of GH, and can stimulate IGF-IR to promote CLL cell survival. Moreover, a recent study showed that the high expression of *IGF-IR* gene in CLL was associted with bad prognostic indicators including *IGHV* gene unmutated status, high CD38 expression, trisomy 12, and del(11)(q23) [161]. Furthermore, the increae in *IGF-IR* gene expression charectierized a subgroup of CLL patients with *NOTCH1* mutation. In a separate study, 3 different IGF-IR inhibitors reduced CLL cell viability and induced apoptosis, even in the presence of the surrounding stromal cells, which provide a protective barrier [24]. Furthermore, it was found that sorafenib, a multikinase inhibitor, was able to decrease IGF-IR expression as well as decrease tyrosine kinase activity, suggesting IGF-IR is a target for sorafenib in CLL.

Significant work has also been accompalished in T-ALL and B-ALL to study the effects of not only IGF-IR but also IGFs. For instance, one study reported the characteristics of insulin, IR, IGF-I, IGF-II, and GH binding in 18 established T-ALL and B-ALL cell lines [162]. IGF-IR and IR were present in these cell lines; IGF-IR was preferentially expressed in T-ALL cell lines and IR was preferentially expressed in B-ALL cell lines. In addition, using binding assays, it was determined that there were different binding affinities for IGF-I, IGF-II, and insulin in the different cell lines. This was the first report to document the expression of these proteins in ALL. Another *in vitro* study used FL5.12, a murine pro B-ALL cell line, to further elucidate the main mechanism of IGF-IR signaling in this disease [163]. This cell line contains a Y1250F/Y1251F double-mutant IGF-IR, which causes loss of apoptotic induction in response to IGF-I withdrawal and deficient colony-forming capabilities and metastatic potential; however, these cells still display mitotic activity [163–166]. This study showed that the FL5.12 cells had impaired phosphorylation of JNK, SHC, and MAPK upon IGF-I stimulation, but IGF-I-induced phosphorylation of AKT was comparable to that in wild-type cells. Thus, this mutation hinders IGF-IR signaling by specifically abrogating the JNK, SHC, and MAPK pathways [163].

Some studies have aimed at identifying potential causes of IGF-IR overexpression in ALL. At least two groups have identified deregulated microRNAs that contribute to IGF-IR overexpression in T-ALL. It was shown that Notch1 repressed the expression of microRNA-223, which, in turn, was able to decrease the expression IGF-IR protein. However, increased expression of microRNA-233 alone did not significantly hinder cell growth, alluding to the possibility that, in addition to the decrease in IGF-IR expression, modifications of other survival molecules are probably required to fully suppress T-ALL [167]. Another study showed that the forced expression of microRNA-99a and microRNA-100 inhibited the expression of IGF-IR and mTOR, as well as the downstream oncogenic proteins MCL1 and the FK506-binding protein 51 in T-ALL cell lines. Importantly, microRNA-99a and microRNA-100 were significantly decreased in 111 ALL patients, and their decrease correlated with poor survival [168].

Evidence based on *in vitro* studies showed that targeting IGF-IR is a potential therapeutic strategy in ALL. For instance, one study showed that PPP efficiently decreased cell viability and induced G_2_/M-phase cell cycle arrest in Jurkat and Molt-3 T-ALL cell lines, with no effect on normal T lymphocytes. PPP was also able to increase the expression of cleaved caspase 3 and PARP and downregulate the anti-apoptotic proteins BCL-2 and BCL-X_L_, signifying apoptotic cell death. Furthermore, PPP decreased the phosphorylation of AKT and MAPK in T-ALL cells [64].

### Myeloproliferative neoplasms (MPN) other than CML

This category encompasses a relatively uncommon group of hematological neoplasms that have specific clinicopathological and molecular characteristics. Polycythemia vera (PV), primary myelofibrosis, and essential thrombocythemia are the most frequently encountered MPN.

The role of IGF/IGF-IR signaling in MPN has been investigated in a limited number of studies, and most of these studies focused on PV. An earlier study showed that the early circulating erythroid progenitors collected from PV patients and maintained in serum-free medium possess remarkably enhanced sensitivity to IGF-I, much more pronounced than the sensitivity of control progenitor cells from healthy subjects, attesting to probable contribution of IGF-I/IGF-IR signaling to PV pathogenesis. Notably, the sensitivity of the cells from PV patients to IGF-I substantially exceeded their sensitivity to erythropoietin [169]. To investigate the molecular basis of IGF-I hypersensitivity, the same group found that basal and IGF-I-stimulated levels of IGF-IRβ subunit's tyrosine phosphorylation were more enhanced in circulating mononuclear cells from patients with PV than in cells from normal individuals [170]. Furthermore, IGFBP-1 was shown to be significantly elevated in PV patients. Functionally, IGFBP-1 from these patients had pronounced stimulatory effects on erythroid burst formation *in vitro* [171]. Subsequent contradictory data showed no differences in IGF-IR phosphorylation levels in PV patients versus healthy controls [172]. However, this latter study further supported alterations in IGFBP levels including increased IGFBP-1, -2, and -4, and decreased IGFBP-3 in these patients. More recently, the expression of *JAK2^V617F^* mutant, a hallmark molecular event in PV, was found to render Ba/F3 cells hypersensitive to IGF-I stimulation, providing a molecular evidence that connects IGF-I/IGF-IR signaling to PV [173].

Arsenite-inducible RNA-associated protein-like (AIRAPL) is an evolutionary conserved regulator of cellular proteostasis in nematodes. A recent study demonstrated that AIRAPL-deficient mice develop a fully penetrant MPN-like disease [174]. Further analysis of the mechanisms underlying these findings demonstrated that AIRAPL promotes the ubiquitination and proteasome degradation of newly synthesized IGF-IR receptor polypeptides, which leads to apoptotic cell death. The lack of AIRAPL and its inhibitory effects on IGF-IR signaling and apoptosis, in reverse, lead to the development of MPN. This intriguing idea was further supported by: (1) targeting IGF-IR prevented MPN in AIRAPL-deficient mice as well as in mice carrying the *Jak2^V617F^* mutation; and (2) the expression of AIRAPL was found to be commonly lacking in human MPN samples [174].

### Malignant lymphoma

The potential contribution of IGF-IR to the pathogenesis of the different subtypes of malignant lymphoma has not been sufficiently investigated. Some studies, however, have shown that IGF-IR is over-expressed in some types of lymphoma cells [22, 23, 175, 176]. Studies from our lab supported a role for IGF-IR in nucleophosmin-ALK-expressing (NPM-ALK^+^) T-cell lymphoma [22]. In this regard, IGF-IR and IGF-I are widely overexpressed in NPM-ALK^+^ T-cell lymphoma cell lines and in ALK^+^ lymphoma tumors from patients [22]. We were also able to identify novel reciprocal functional interactions between IGF-IR and NPM-ALK that were demonstrated through transfection experiments using wild-type and mutated constructs of IGF-IR and NPM-ALK [22, 65]. Transfection of an NPM-ALK^+^ T-cell lymphoma cell line with wild-type NPM-ALK significantly increased pIGF-IR, and transfection with wild-type IGF-IR induced a marked increase in pNPM-ALK [22, 65]. Transfection of non-functional mutants failed to induce similar effects. Our studies also showed that selective antagonism of IGF-IR with PPP decreased cell viability, induced apoptosis and G_2_/M-phase cell-cycle arrest, and decreased proliferation and colony formation of NPM-ALK^+^ T-cell lymphoma cells [22]. In a more recent work, similar findings were observed when GSK1838705, a small molecule IGF-IR/IR/ALK inhibitor, was utilized to treat a variety of different cancers with high expression of IGF-IR, including NPM-ALK^+^ T-cell lymphoma [177].

**Figure 4:**
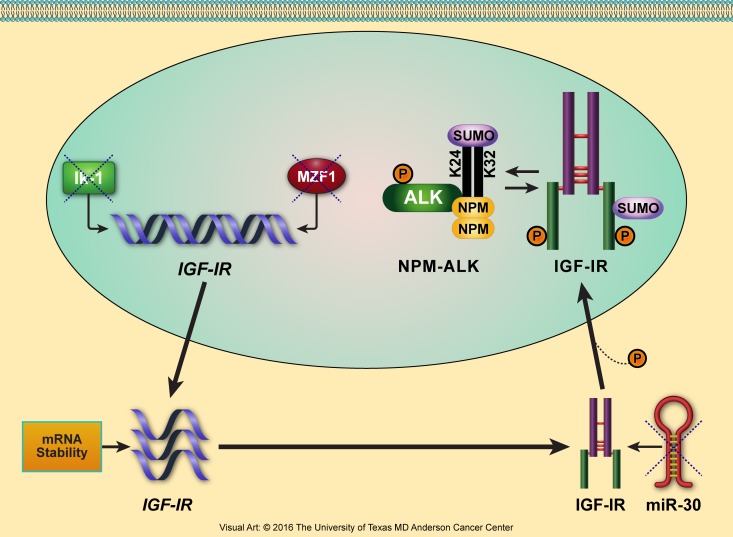
Proposed model illustrating the multilevel deregulatory mechanisms that lead to upregulation of IGF-IR expression in NPM-ALK^+^ T-cell lymphoma We have been using NPM-ALK^+^ T-cell lymphoma as a cancer model to identify novel mechanisms underlying upregulation of IGF-IR expression. The levels of the transcription factors Ik-1 and MZF1 are significantly decreased in this lymphoma, preventing them from transcriptionally inhibiting *IGF-IR* gene expression and allowing *IGF-IR* mRNA to be transcribed. Furthermore, slower decay of *IGF-IR* mRNA increases its bioavailability for protein translation. In addition, microRNA-30a and microRNA-30d are also markedly decreased in NPM-ALK^+^ T-cell lymphoma (unpublished data). Lack of suppression of the IGF-IR-3′-UTR by these microRNAs leads to the posttranscriptional stabilization and upregulation of IGF-IR protein. IGF-IR protein is further stabilized by the posttranslational modification SUMOylation. SUMOylated IGF-IR can indirectly stabilize NPM-ALK, which is also capable of being SUMOylated [303].

Furthermore, in our lab, we used NPM-ALK^+^ T-cell lymphoma as a cancer model to identify novel transcriptional and posttranscriptional mechanisms underlying the aberrant increase in IGF-IR expression (Figure 4). For instance, the transcription factors Ikaros isoform-1 (Ik-1) and myeloid zinc finger 1 (MZF1) are markedly decreased in this lymphoma. Importantly, we found that Ik-1 and MZF1 are capable of down-regulating *IGF-IR* gene expression by binding directly to its promoter and 5′ region [178]. As result of decreasing *IGF-IR* mRNA and protein expression, both Ik-1 and MZF1 also decreased the expression of phosphorylated IGF-IR, which subsequently decreased the phosphorylation of important downstream signaling proteins such as AKT and IRS-1. Ectopic expression of these transcription factors decreased lymphoma cell viability, proliferation, migration, and colony-forming potential. We also demonstrated that the decay of *IGF-IR* mRNA is significantly delayed in NPM-ALK^+^ T-cell lymphoma cell lines compared with normal T lymphocytes, suggesting a second possible mechanism by which IGF-IR is upregulated in these cells [178].

To a lesser extent, a role of GH in regulating IGF-IR expression has been demonstrated in mouse T-cell EL4 lymphoma cells. It was shown that one of the consequences of overexpression of endogenous GH was an increase in the expression of IGF-I and IGF-IR, which mediated the protection of these lymphoma cells from apoptosis. As mentioned previously, GH regulates the secretion of IGF-I by the liver, although IGF-I concentrations can arise independently of GH. The EL4 lymphoma model may demonstrate a potential method of upregulation of IGF-IR via GH [175].

Our group has also demonstrated overexpression of IGF-IR in mantle cell lymphoma (MCL) cell lines and human lymphoma tissues [23]. The inhibition of IGF-IR by siRNA and PPP downregulated pIRS-1, pAKT, and pSTAT3 and induced caspase-3 cleavage in MCL. To our knowledge, these results were the first to report the expression of IRS-1 and pIRS-1 in MCL, which suggests that these proteins, through interactions with IGF-IR, could contribute to the survival of this aggressive lymphoma.

In Hodgkin lymphoma (HL), a recent study demonstrated that IGF-IR was overexpressed in 55% of the patients, and pIGF-IR was detectable in only 3 of the 16 IGF-IR-positive tumors that were analyzed for pIGF-IR expression [179]. *In vitro* experiments showed that the mitotic potential of HL cells is highly dependent on IGF-I, as treatment with the ligand resulted in increased phosphorylation of IGF-IR, AKT, and ERK in L428 and L1236 HL cells. Treatment with PPP was able to induce cell growth inhibition via G_2_/M-phase cell cycle arrest. In addition, positive IGF-IR status correlated with higher overall survival and 5-year progression-free survival rates compared with IGF-IR-negative patients, suggesting that positive IGF-IR status is in fact a prognostic marker for HL patients. This same phenomenon was also shown in a separate study, in which IGF-IR was associated with a better prognosis in patients with classical HL. However, this study also suggested that IGF-IR expression was closely associated with MET receptor expression, which allows another mode of therapeutic interference [180]. Despite the widely documented cancer-promoting effects of IGF-IR signaling, these two studies failed to provide an explanation for the better outcome of HL patients with IGF-IR expression in their tumors.

Diffuse large B-cell lymphoma (DLBCL) occurs in adults, and the standard chemotherapy treatment results in improvement in 60-70% of patients. Nonetheless, other treatment options are being sought for those in whom standard chemotherapy has failed. At least two IGF-IR inhibitors have been shown to efficiently induce cellular death in DLBCL *in vitro:* PPP and NVP-AEW541 [181]. As in other studies using PPP, DLBCL cells underwent apoptosis upon treatment with this inhibitor, proliferation was reduced, and cell growth was hindered through a G_2_/M-phase cell cycle arrest with complete elimination of cells in the G_0_/G_1_ phase of the cell cycle. Interestingly, however, PPP did not decrease the phosphorylation of IGF-IR at Tyr1131/Tyr1136 or its downstream targets AKT and ERK1/2, suggesting another possible mechanism of action in these cells. In contrast, NVP-AEW541 significantly inhibited phosphorylation of IGF-IR at Tyr1131/Tyr1136 and AKT, while ERK1/2 phosphorylation was less affected, suggesting that dual treatment with these inhibitors might provide more effective therapy for DLBCL [181].

## PRECLINICAL AND CLINICAL EVALUATION OF IGF-IR INHIBITORS AND MECHANISMS OF RESISTANCE TO THESE INHIBITORS

One of the reasons why IGF-IR was initially considered a poor choice therapeutic option was the fact that it shares a high percentage of amino acid sequence identity with IR. However, more recently, there have been several approaches to target IGF-IR more specifically. Strategies to inhibit IGF-IR signaling in human cancers include: (1) monoclonal antibodies that directly block IGF-IR; (2) monoclonal antibodies that function by neutralizing the circulating ligands IGF-I or IGF-II, which prevents IGF-IR from being activated; and (3) small molecule inhibitors that antagonize the kinase activity of IGF-IR [182]. The anti-IGF-IR monoclonal antibodies sometimes result in insulin resistance, hyperinsulinemia, and hyperglycemia. Similar to IGF-IR monoclonal antibodies, the primary side effect of small molecule inhibitors is hyperglycemia, possibly through the inhibition of IR [183–187]. Table 1 lists the IGF-IR inhibitors that have been evaluated in preclinical studies. In addition, Table 2 lists the inhibitors that have been utilized in clinical trials, with different hematological neoplasms highlighted in RED.

**Table 1:** Inhibitors of IGF-IR that underwent pre-clinical evaluation

Inhibitor	Phase	Tumor Type	Manufacturer	Type of inhibitor	References
A-928605	Pre-clinical	Neuroblastoma	Abbott	Dual IGF-IR/IR tyrosine kinase inhibitor	[283]
ABDP	Pre-clinical	Colon cancer	AstraZeneca	Dual IGF-IR/IR tyrosine kinase inhibitor	[284]
AG-1024	Pre-clinical	Breast cancer, colorectal cancer, glioma, hepatocellular carcinoma, mesothelioma, NSCLC, osteosarcoma, pancreatic cancer, prostate cancer	Calbiochem EMD Biosciences	Tyrosine kinase and autophosphorylation inhibitor	[268, 285–294]
BMS-536924	Pre-clinical	AML, breast cancer, ovarian cancer	Bristol-Myers Squibb	ATP-competitive inhibitor	[59, 249, 295]
BMS-554417	Pre-clinical	Breast cancer, ovarian cancer	Bristol-Myers Squibb	Reversible ATP-competitive small molecule inhibitor	[153]
GSK1838705A	Pre-clinical	Breast cancer, cervical cancer, colon cancer, Ewing sarcoma, glioma, head and neck cancer, hepatocellular carcinoma, lung cancer, NPM-ALK^+^ T-cell lymphoma, ovarian cancer, PCM, pancreatic cancer, prostate cancer	GlaxoSmithKline	Triple IGF-IR/IR/ALK tyrosine kinase inhibitor	[177, 296–298]
GSK1904529A	Pre-clinical	Askin tumor, breast cancer, cervical cancer, colon cancer, Ewing sarcoma, head and neck cancer, lung cancer, NPM-ALK^+^ T-cell lymphoma, ovarian cancer, PCM, prostate cancer	GlaxoSmithKline	Dual IGF-IR/IR tyrosine kinase inhibitor	[299]
INSM-18	Pre-clinical	Prostate cancer	Insmed	Dual IGF-IR/HER2 small molecule inhibitor	[300]
NVP-ADW742	Pre-clinical	AML, Ewing sarcoma, medulloblastoma, NSCLC	Novartis	Reversible ATP-competitive small molecule inhibitor	[60, 257, 259, 301, 302]
NVP-AEW541	Pre-clinical	AML, breast cancer, colon cancer, endometrial cancer, esophageal cancer, Ewing sarcoma, gastric cancer, gastrointestinal stromal tumors, glioma, head and neck cancer, hepatocellular carcinoma, medulloblastoma, neuroblastoma, ovarian cancer, pancreatic cancer, PCM, pituitary tumors, prostate cancer, soft tissue sarcomas, synovial sarcoma	Novartis	Reversible ATP-competitive small molecule inhibitor	[56, 57, 61, 218–240]
Picropodophyllin (PPP; AXL1717)	Pre-clinical	Breast cancer, CML, colon cancer, Ewing sarcoma, glioblastoma, hepatocellular carcinoma, lung cancer, MCL, medulloblastoma, neuroblastoma, NPM-ALK^+^ T-cell lymphoma, osteosarcoma, ovarian cancer, pancreatic cancer, PCM, uveal melanoma	Axelar	Non-ATP-competitive small molecule inhibitor	[21–23, 56, 64, 203–216]

**Table 2:** IGF-IR inhibitors that were used in clinical trials

Inhibitor	Phase	Tumor Type	Manufacturer	Type of Inhibitor	Web links and references
AMG 479 (Ganitumab)	Phase I/II	Breast cancer, colorectal cancer, Ewing sarcoma, lung cancer, lymphoma, melanoma, ovarian cancer, pancreatic cancer, refractory solid tumors, soft tissue sarcomas	Amgen	Monoclonal antibody (IgG_1_)	https://clinicaltrials.gov/ct2/results?term=AMG-479&pg=2
AVE1642	Phase I/II	Breast cancer, liver cancer, PCM	Sanofi-Aventis	Monoclonal antibody (IgG_1_)	https://clinicaltrials.gov/ct2/results?term=AVE1642&Search=Search
BIIB022	Phase I	Hepatocellular carcinoma, NSCLC, refractory solid tumors	Biogen	Monoclonal antibody (IgG_4_)	https://clinicaltrials.gov/ct2/results?term=BIIB022&Search=Search
BMS-754807	Phase I/II	Advanced or metastatic solid tumors, breast cancer, colorectal carcinoma, head and neck squamous cell carcinoma	Bristol-Myers Squibb	Dual IGF-IR/IR tyrosine kinase inhibitor-reversible ATP competitive inhibitor	https://clinicaltrials.gov/ct2/results?term=BMS-754807&Search=Search
CP-751,871 (Figitumumab)	Phase I/II/III	Advanced solid tumors, breast cancer, colorectal cancer, Ewing sarcoma, head and neck squamous cell carcinoma, lung cancer, PCM, prostate cancer	Pfizer	Monoclonal antibody (IgG_2_)	https://clinicaltrials.gov/ct2/results?term=CP-751%2C871&Search=Search
IMC-A12 (Cixutumumab)	Phase I/II	Adrenocortical carcinoma, brain stem neoplasms, breast cancer, ciliary body and choroid melanoma, colorectal cancer, esophageal carcinoma, Ewing sarcoma, head and neck squamous cell carcinoma, hepatocellular carcinoma, lung cancer, mesothelioma, neuroendocrine neoplasms, osteosarcoma, prostate cancer, recurrent or refractory solid tumors, soft tissue sarcomas, thymoma and thymic carcinoma	ImClone	Monoclonal antibody (IgG_1_)	https://clinicaltrials.gov/ct2/results?term=IMC-A12&Search=Search
MK-0646 (Dalotuzumab)	Phase I/II	Advanced solid tumors, breast cancer, colorectal cancer, lung cancer, neuroendocrine neoplasms, ovarian cancer, pancreatic cancer, PCM	Merck	Monoclonal antibody (IgG_1_)	https://clinicaltrials.gov/ct2/results?term=MK-0646&Search=Search
OSI-906 (Linsitinib)	Phase I/II/III	Advanced solid tumors, adrenocortical carcinoma, breast cancer, colorectal cancer, Ewing sarcoma, gastrointestinal stromal tumors, head and neck squamous cell carcinoma, hepatocellular carcinoma, lung cancer, ovarian cancer, pancreatic cancer, PCM, prostate cancer	OSI Pharmaceuticals	Reversible ATP-competitive small molecule inhibitor	https://clinicaltrials.gov/ct2/results?term=OSI-906&Search=Search
Picropodophyllin (PPP; AXL1717)	Phase I/II	Malignant astrocytomas, NSCLC	Axelar	Non-ATP-competitive small molecule inhibitor	https://clinicaltrials.gov/ct2/results?term=Picropodophyllin+&Search=Search and [217]
R1507	Phase I/II	Breast cancer, Ewing sarcoma, NSCLC, osteosarcoma, soft tissue sarcomas	Roche	Monoclonal antibody (IgG_1_)	https://clinicaltrials.gov/ct2/results?term=R1507&Search=Search
SCH717454 (Robatumumab)	Phase I/II	Advanced solid tumors, colorectal cancer, Ewing sarcoma, neuroblastoma, osteosarcoma, pediatric solid tumors, soft tissue sarcomas	Schering-Plough	Monoclonal antibody (IgG_1_)	https://clinicaltrials.gov/ct2/results?term=SCH717454&Search=Search
XL-228	Phase I	Advanced solid tumors, CML, lymphoma, PCM, Ph+ ALL	Exelixis	Dual IGF-IR/SRC small molecule inhibitor	https://clinicaltrials.gov/ct2/results?term=XL-228&Search=Search

An example of the anti-IGF-IR blocking antibodies is AVE1642, which had encouraging effects *in vitro* and *in vivo* in animal models in different types of cancer including lung, hepatocellular carcinoma, PCM, Ewing sarcoma, and neuroblastoma, as well as against breast cancer metastasis [188–191]. Furthermore, it was well tolerated as a single agent or in combination with other chemotherapeutics in early clinical trials [192–194]. In addition, the IMC-A12 (cixutumumab) anti-IGF-IR monoclonal antibody achieved cancer growth inhibition *in vitro* and in xenograft models [195–198]. The efficacy of this antibody has since been evaluated in clinical trials, alone or in combination with other therapeutic agents [183–185, 199–202]. In addition to AVE1642 and cixutumumab, several other anti-IGF-IR monoclonal antibodies underwent preclinical and clinical trials evaluations, and are listed in Tables 1 and 2, respectively.

The IGF-IR small molecule inhibitors compete for the binding site for ATP within the IGF-IR kinase domain. For example, in *in vitro* and *in vivo* preclinical models, PPP was shown to demonstrate pronounced inhibitory effects on cancer cells, including growth inhibition, cell cycle arrest, and apoptosis [21–23, 56, 64, 203–216]. Because of promising preclinical outcomes, the effects of PPP have also been explored in clinical trials [217]. NVP-AEW541 is another IGF-IR small molecule inhibitor that has been extensively tested in *in vitro* and *in vivo* preclinical studies in a variety of cancer cell types [56, 57, 61, 218–240]. Some of the small molecule inhibitors of IGF-IR that underwent preclinical evaluations are listed in Table 1, and, in addition, some of the ones tested in clinical trials are shown in Table 2.

Even when IGF-IR inhibitors were tolerated, significant number of patients did not exhibit improved outcomes. While further evaluations are required to characterize the mechanisms behind the shortcomings of these inhibitors, several studies underscored that an adequate number of IGF-IR receptors per cell or high levels of expression of IGF-IR protein or mRNA is required to achieve a robust response to the inhibitors [241–245]. Although an inherent low number of IGF-IR molecules per cancer cell may cause primary resistance to anti-IGF-IR antibodies or small molecule inhibitors, this factor appears to be more relevant to inducing secondary resistance when anti-IGF-IR antibodies are used. After the anti-IGF-IR antibody/IGF-IR complex undergoes endocytosis and subsequent degradation, a substantial reduction in the number of IGF-IR molecules on the surface of the cells typically occurs, which could cause secondary resistance after an initial response to the effects of IGF-IR antibodies [244]. The resistance to IGF-IR inhibitors can also stem from other members of the IGF system receptors including the IR, particularly IR-A and to a lesser extent IR-B [244, 246–248]. Moreover, the hybrid receptor IGF-IR/IR-A has also been suggested to induce significant resistance to IGF-IR inhibitors.

**Figure 5:**
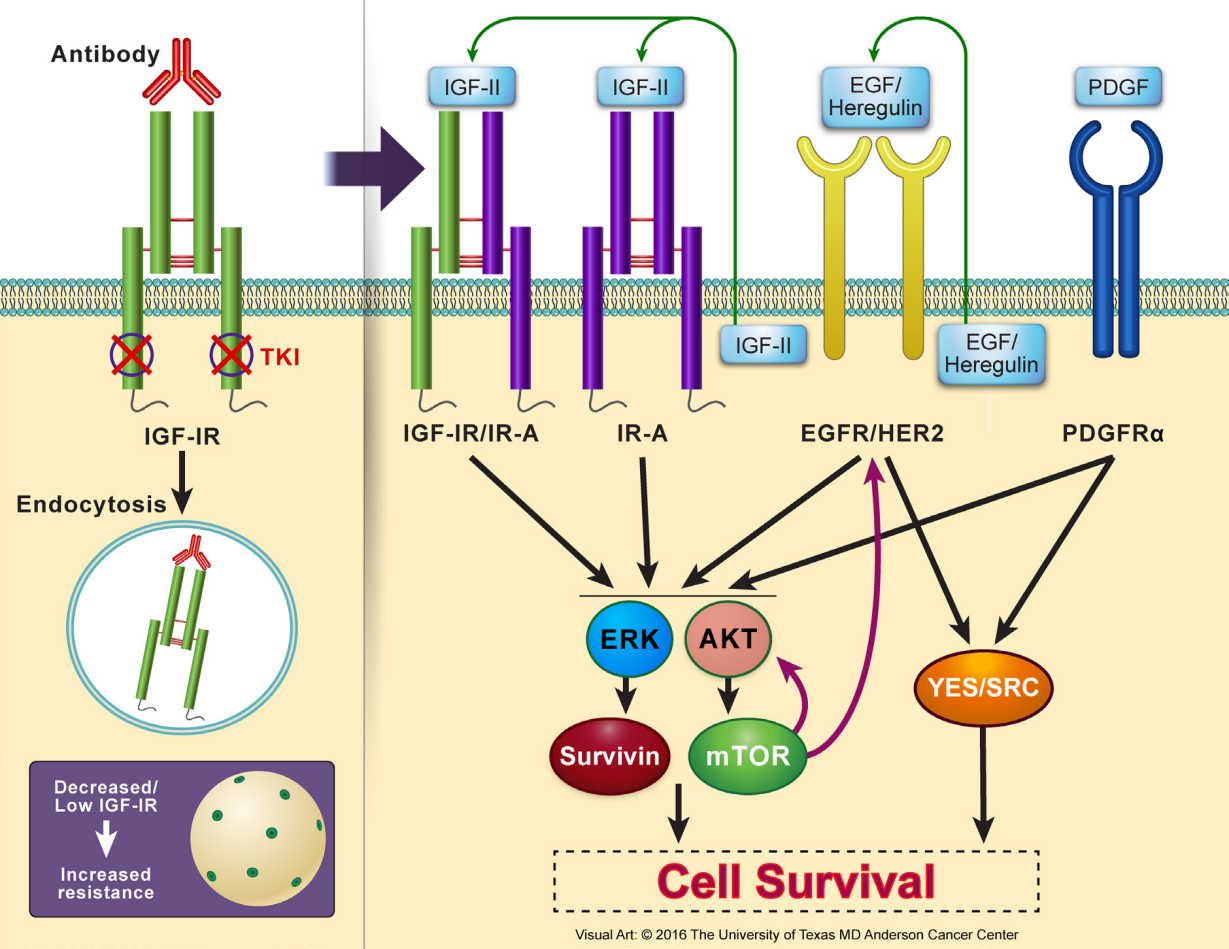
Resistance to IGF-IR inhibition in cancer cells Several resistance mechanisms to IGF-IR inhibitors have been reported using *in vitro* and *in vivo* experimental models as well as proposed in clinical trials. Lack of abundant levels of IGF-IR protein expression or adequate number of IGF-IR receptor molecules per cell, which could be an inherent criterion of a specific type of cancer or acquired after initiation of IGF-IR inhibition therapy particularly with using IGF-IR antibodies that cause endocytosis and degradation of IGF-IR, is believed to be an important factor leading to resistance. Resistance to IGF-IR inhibition can be mediated through other members of the IGF system receptors that possess oncogenic potential such as IR-A or the hybrid receptor IGF-IR/IR-A. In addition, resistance can also be initiated through other non-IGF signaling systems. Examples of these include EGFR/HER2 and PDGFRα. Collectively, resistance systems bypass IGF-IR inhibition by providing alternate downstream activation of AKT and MAPK. These kinases further activate/sustain mTOR and survivin. mTOR, in return, contributes through a feedback loop to upregulation of EGFR/HER2 and AKT. EGFR/HER2 and PDGFRα can also surpass the effects of IGF-IR inhibition by activating the SRC kinases family. Additional studies are still required to further analyze the mechanisms that cause resistance to IGF-IR inhibitors, which is a very important limiting factor to the wide clinical utilization of these inhibitors to treat cancer patients. These studies may also shed light on mechanisms of resistance to other targeted therapeutics.

One of the widely studied mechanisms for cancer cell resistance to IGF-IR inhibition is the upregulation of AKT/mTOR signaling pathway. It was shown that inhibition of IGF-IR induces activation of AKTmTOR, resulting in a feedback synthesis and activation of AKT as well as of epidermal growth factor receptor (EGFR) family and survivin. These proteins possess pronounced ability to overcome the effects of IGF-IR inhibitors and cause survival of the resistant cells [32, 234, 236, 241, 249–252]. Similarly, potentiation of ERK/MAPK signaling has been implicated in the resistance to IGF-IR inhibitors [32, 238, 244, 253]. Also, recent studies suggested that a bypass activation of SRC kinases family induces significant resistance to IGF-IR inhibitors in cancer cells [254]. Activated SRC acts through integrin α5β3, which leads to considerable resistance to IGF-IR inhibition [255]. Signaling through platelet-derived growth factor receptor-α (PDGFRα) has also been proposed to enhance the resistance to targeting IGF-IR [237, 243]. In support of this notion, PDGFRα is overexpressed and constitutively activated in cells resistant to IGF-IR inhibitors. Figure 5 illustrates some of the most commonly proposed mechanisms of resistance to IGF-IR inhibitors.

## IGF-IR SIGNALING INDUCES RESISTANCE TO DIFFERENT CANCER THERAPEUTIC MODALITIES

The IGF-IR-related drug resistance in cancer does not only result from the utilization of IGF-IR inhibitors. In fact, IGF-IR signaling, by itself, can elicit substantial resistance to other cancer therapeutic modalities including chemotherapy and radiotherapy [256–263]. Moreover, IGF-IR has been shown to induce resistance to agents that selectively target oncogenic molecules other than IGF-IR. In a fashion reminiscent to the effects of EGFR family of kinases on inhibitors of IGF-IR, substantial evidence from several laboratories supported that IGF-IR signaling causes resistance to the blocking antibodies and small molecule inhibitors that interfere with the signaling of EGFR/HER2 kinases family [264–270]. This phenomenon has been reported in a variety of cancers including those of the lung, breast, and prostate. It is possible that heterodimerization between IGF-IR and EGFR/HER2 contributes to the resistance to EGFR inhibitors [271, 272]. In addition to resistance to the inhibition of EGFR, IGF-IR has been shown to induce significant resistance to inhibitors of several other cancer survival signaling including those functioning through modulation of the estrogen and androgen receptors (breast and prostate cancers, respectively), proteasome degradation (PCM), ALK kinase (NSCLC), ATM-related kinase (ATR; breast cancer), and the colony-stimulating factor-1 receptor (CSF-1R; gliomas) [273–279]. Although the mechanisms of IGF-IR-induced resistance are not completely defined, upregulation and bypass activation of survival-promoting proteins have been demonstrated. For example, a recent study showed that in breast cancer cells resistant to the effects of the anti-EGFR/HER2 antibody trastuzumab, IGF-IR induces resistance through the SRC/FAK/FoxM1 signaling [280]. Other studies also showed that IGF-IR-mediated upregulation of MAPK/ERK signaling could be another mechanism for drug resistance [275]. But one of the most frequently implicated modes of cancer cell resistance is the ability of IGF-IR to enhance PI3K/AKT/mTOR signaling, which could bypass the effects of the therapeutic agents. This mechanism has been demonstrated in different types of cancer cells including breast cancer, NSCLC, and head and neck squamous cell carcinoma cells, and by using different types of cancer antagonists including hormonal modulation, chemotherapeutic agents, EGFR small molecule inhibitors gefitinib and erlotinib, and AKT small molecule inhibitor AZD5363 [258, 264, 272, 275, 276]. IGF-IR-induced upregulation of PI3K/AKT/mTOR appears to induce its effects, at least in part, through upregulation of survivin expression [258, 267, 272].

Analyzing carefully the different routes of IGF-IR-induced drug resistance can possibly shed some light on how to intervene with novel therapeutics to overcome this setback. It is important to note that the mechanisms by which resistance to IGF-IR inhibitors evolve are largely similar to the mechanisms induced by IGF-IR against other therapeutic modalities. Therefore, it is not surprising that preclinical studies and clinical trials where combined targeting of IGF-IR and other survival promoting proteins that reciprocally interact with IGF-IR to cause drug resistance provided promising results implicating that such an approach could be more beneficial than using one agent alone [201, 258, 264, 272, 277, 278, 279, 280, 281, 282]

## CONCLUSIONS

In spite of the breakthroughs that have occurred in treating patients with hematological malignancies, important gaps in knowledge still exist in understanding the pathobiology of these aggressive neoplasms. Unraveling the mysteries underlying these gaps is expected to further facilitate the development of novel therapeutic strategies to tackle and eradicate these neoplasms. Although a relatively fewer studies have analyzed the role of IGF-IR in hematological neoplasms than in solid cancers, strong evidence suggests that this oncogenic receptor may become a useful target, alone or in combination, for treating hematological neoplasms.

Several *in vitro* and *in vivo* experimental models have provided links between IGF-IR signaling and downstream mediators that maintain the survival of PCM, leukemia, and lymphoma. Table 3 summarizes some of these mechanisms. Characterizing the effects of IGF-IR has led to the development of promising targeting approaches, including monoclonal antibodies and small molecule inhibitors that have shown potent antitumor activity *in vitro* and in animal models.

**Table 3:** Summary of mechanistic roles of IGF-IR in the pathogenesis of hematological malignancies

Disease Type	Major Pathways Involved	Mechanistic Role of IGF-IR	Outcome
PCM	PI3K/AKT: inhibition of apoptosis through the inhibition of cytochrome C release from mitochondria MAPK: induction of cellular proliferation PI3K/AKT/MAPK crosstalk: PI3K can directly phosphorylate MEK1/2 and MAPK	IGF-I independent mechanism: direct phosphorylation of IRS molecules on IGF-IR, mainly IRS-2 IGF-I/IL-6 dual autocrine/paracrine loops contribute to PCM cells growth and survival through IGF-IR IGF-I dependent mechanism: IGF-I binding to IGF-IR, causing autophosphorylation of tyrosine residues and initiating downstream pathways IGF-II induced protection from apoptosis IGF-I induced inhibition of PTEN	Effects on apoptosis and cellular proliferation
	mTOR-dependent serine phosphorylation of IRS-1 enhances IGF-IR signaling and PI3K/AKT activation	IGF-I as a chemoattractant increases adhesion of PCM cells to fibronectin when PI3K/AKT is activated IGF-I induced association of IGF-IR and beta-1 integrin through the phosphorylation of IGF-IR, IRS-1, and PI3K IGF-I induced polymerization of F-actin, phosphorylation of FAK protein and paxillin, and enhance beta-1 integrin with focal adhesion proteins during homing process IGF-I increases migration of PCM cells in an AKT-dependent or -independent manner (such as PI3K-PKD or PI3K-RhoA pathways)	Effects on adhesion, invasion, and migration
Leukemia	PI3K/AKT and mTOR pathways are activated in immature AML cells Constitutive activation of AKT/PKB in AML blasts due to autocrine IGF-I/IGF-IR signaling	IGF-I induced drug resistance in AML Inhibition of apoptosis	Effects in AML
	Pathway crosstalk: overactivation of PI3K/AKT via the upregulation of IRS-2 and IGF-IR autocrine signaling or through mTOR inhibition IGF-IR induced activation of JAK/STAT pathway IGF-IR induced upregulation of BCL-2, BCL-X_L_ and inhibition of caspase activation	Induction of cellular proliferation through deregulation of cell cycle molecules such as cyclin B1, cyclin E, and pCdc2 c-myb induced overexpression of IGF-I, IGF-II, and IGF-IR and a decrease in IGFBP-3	Effects in CML
	c-myb induced IGF-IR activation and downstream AKT and ERK pathways		
	Upregulation of IGF-IR and BCL-2	Increased local production of serum IGF-I, independently of GH, controls autocrine/paracrine stimulation of IGF-IR	Effects in CLL
	IGF-IR impairs JNK, SHC, MAPK in B-ALL Y1250F/Y1251F mutant cells; IGF-I stimulation induced phosphorylation of AKT in the mutant cells, suggesting the Y1250F/Y1251F mutations can only hinder JNK, SHC, and MAPK pathways IGF-IR inhibition decreases PI3K/AKT, MAPK pathways while decreasing BCL-2 and BCL-X_L_and increases cleaved caspase 3 and PARP	IGF-IR is preferentially expressed in T-ALL cell lines MicroRNA-223 decreases IGF-IR expression in T-ALL MicroRNA-99a and microRNA-100 overexpression inhibits the expression of IGF-IR and mTOR, as well as downstream proteins MCL-1 and FKBP51 in T-ALL	Effects in T-ALL Effects in B-ALL
MDS		IGF-IR induced transformation of MDS to AML IGF-IR induced protection of apoptosis due to overexpression in advanced MDS subtypes	Effects in MDS
Lymphoma	IGF-IR induced activation of PI3K/AKT and JAK/STAT pathways	Novel functional interactions between IGF-IR and NPM-ALK in NPM-ALK^+^ T-cell lymphoma Inhibition of apoptosis Induction of cellular proliferation Ik-1 and MZF1 transcriptionally decrease the expression of *IGF-IR* in ALK^+^ T-cell lymphoma mRNA decay is significantly hindered in ALK^+^ T-cell lymphoma cells	Effects in NPM-ALK^+^ T-cell lymphoma
	IGF-IR induced phosphorylation of IRS-1 in mantle cell lymphoma IGF-IR inhibition resulted in hindrance of the PI3K/AKT and JAK/STAT pathways, and increased the expression of cleaved caspase 3 and PARP	Inhibition of apoptosis and induction of cellular proliferation	Effects in MCL
	IGF-I induced PI3K/AKT and ERK pathways IGF-IR/MET receptor interaction	The mitotic potential of HL cells is highly IGF-I dependent	Effects in HL
	Inhibition of IGF-IR reduces activation of PI3K/AKT and ERK pathways	HL cells underwent apoptosis upon IGF-IR inhibition and proliferation was reduced; cell growth was hindered through a G_2_/M-phase cell cycle arrest with complete elimination of cells in the G_0_/G_1_ phase of the cell cycle.	Effects in DLBCL
	GH signaling	Overexpression of endogenous GH leads to an increase in the expression of IGF-I and IGF-IR, which protects lymphoma cells from apoptosis	Effects in mouse EL4 lymphoma

Although early evidence at least from some clinical trials suggested that targeting IGF-IR could represent a promising approach to treating cancer patients, the outcomes of these trials have not been as conclusive as the *in vitro* and animal research. We believe that one relevant explanation for this discrepancy is that the selection of patients included in the trials was mostly random and not rationally justified. The majority of these trials were phase I that included patients who have already been treated with several approaches that most likely have caused their cancers to undergo important molecular and biological modifications that resulted in resistance to more than one therapeutic modality. Instead, selection of patients to be treated by IGF-IR inhibitors, or similar targeted therapeutics, should be based on rigorous criteria such as proven biomarkers, stringent clinical characteristics, and/or histopathological and molecular assays. As an example, a higher success rate for IGF-IR inhibitors could have been achieved if an accurate estimation of the level of expression of IGF-IR and possibly the expression of pIGF-IR was first performed in the patients' tumors prior to their enrollment in the trials. It is quite reasonable to assume that the presence of abundant expression of IGF-IR could positively impact the response to its inhibitors. Utilizing such strategies may improve the clinical outcomes of targeted therapeutics, including IGF-IR inhibitors, in cancer and, hopefully, in time eliminate this aggressive disease.
